# First report of an egg-predator nemertean worm in crabs from the south-eastern Pacific coast: *Carcinonemertes camanchaco* sp. nov

**DOI:** 10.1038/s41598-021-98650-0

**Published:** 2021-10-12

**Authors:** Natalia Verónica Leiva, Luis Ñacari, Juan Antonio Baeza, María Teresa González

**Affiliations:** 1 Instituto de Ciencias Naturales “Alexander von Humboldt”, Facultad de Ciencias del Mar y Recursos Biológicos, Universidad de Antofagasta, 601 Angamos, Antofagasta, Chile; 2 Programa Doctorado en Ciencias Aplicadas, Facultad de Ciencias del Mar y Recursos Biológicos, Universidad de Antofagasta, 601 Angamos, Antofagasta, Chile; 3 Department of Biological Sciences, 132 Long Hall, Clemson University, Clemson, SC 29634 USA; 4 Smithsonian Marine Station at Fort Pierce, 701 Seaway Drive, Fort Pierce, FL 34949 USA; 5 Departamento de Biología Marina, Facultad de Ciencias del Mar, Universidad Católica del Norte, 1281 Larrondo, Coquimbo, Chile

**Keywords:** Zoology, Biodiversity

## Abstract

Nemertean worms belonging to the genus *Carcinonemertes* have been tied to the collapse of crab fisheries in the northeastern Pacific Ocean. A new species is described from egg masses of two commercial crabs, *Cancer porteri* and *Romaleon setosum*, inhabiting the central-north Chilean coast. This is the first species of *Carcinonemertes* described from the southeastern Pacific Ocean. Total body length of *Carcinonemertes camanchaco* sp. nov. ranged from 2.38 to 4.93 and from 4.29 to 8.92 mm, in males and females, respectively. Among others, traits that distinguish this new species from other previously described congeneric species include: presence of two gonad rows on each side of the intestine, a simple (not decorated) mucus sheath, and a relatively wide stylet basis. Maximum likelihood and Bayesian inference phylogenetic analyses distinguished this new species from all other species of *Carcinonemertes* with available *cox1* sequences in GenBank. Prevalence and mean (± SD) intensity of *C. camanchaco* sp. nov. was 24% and 2.6 (± 2.07) worms per egg mass in *C. porteri* and 38.1% and 3.8 (± 2.4) worms per egg mass in *R. setosum*. The formal description of this new species represents the first step towards the understanding of this worm's impact on the health of crab fisheries in the southeastern Pacific Ocean.

## Introduction

Nemerteans belonging to the family Carcinonemertidae are voracious egg-predators that infect a variety of decapod crustaceans^1,2^. Some species are responsible for the collapse of crustacean fisheries in North America^3–6^ given their negative effect on female reproductive performance^2,5,7,8^. Several species of commercial interest, such as the Caribbean spiny lobster *Panulirus argus* whose eggs are consumed by *C. conanobrieni*^9,10^, the Dungeness crab *Metacarcinus magister* infected by *C. errans*^5^, the Red king crab *Paralithodes camtschaticus* parasitized by *C. regicides*, and the sand crab *Portunus pelagicus* infected by *C. mitsukurii*^11^ are used as hosts by carcinonemertid worms. One of the most studied nemertean worms is *C. errans* that during outbreaks causes considerable egg mortality in 50% or more of the brooding female crabs of *Metacarcinus magister*. *Carcinonemertes errans* has been tied to the collapse of this crab fishery in Central California early during the 1960s^1,5,12^.

The family Carcinonemertidae is comprised of two genera, *Carcinonemertes* with sixteen described species, and *Ovicides* with five described species^10^. Members of the genus *Carcinonemertes* have a single stylet and no accessory pouches while the genus *Ovicides* invariably exhibit accessory pouches and more than one stylet. All *Carcinonemertes* are gonochoric (separate sexes) while representatives of *Ovicides* can be either gonochoric or simultaneously hermaphroditic^13,14^. The life cycle of nemertean parasites is variable and appears to be adapted to the reproductive cycle and brooding pattern of their respective crustacean hosts^15^. At one extreme, some nemertean parasites exhibit a rather simple and short life cycle in which the worms feed, mature, and reproduce on a single ovigerous female host. In this case, host autoinfection is common (i.e., *C. regicides* in the Red king crab, *Paralithodes camtschatica*)^15^. At the other extreme, some *Carcinonemertes* are long lived and their life cycle involve the infection of both juvenile (males and females) and adult (males and gravid females) host individuals. In these long lived *Carcinonemertes*, autoinfection is rare and worms are also capable of transferring from male to female hosts when the host crabs mate (i.e., *C. errans* in the Dungeness crab *Metacarcinus magister*)^15^. Host-specificity also varies considerably in the family Carcinonemertidae. Some species of *Carcinonemertes* are specialists, using a single host species^16^ while other species are generalists, such as *C. carcinophila*, reported from as many as 28 different species of decapod hosts^17^.

Currently, 17 species of *Carcinonemertes* are known to science: four species inhabiting the Brazilian coast, South-western Atlantic (C. *carcinophila inminuta*, *C. divae*, *C. caissarum,* and *C. sebastianensis*)^13^, one species from the Caribbean Sea (Florida, USA and Santa Marta, Colombia) and North-western Atlantic Ocean (*C. conanobrieni*)^9,10^, one from the Southwestern Indian Ocean (*C. mitsukurii*, western Australian coast)^18,19^, eight species from the North Pacific Ocean (*C. coei*, *C. errans*, *C. regicides*, *C. epialti*, *C. kurisi*, *C. pinnotheridophila*, *C. c. carcinophila,* and *C. wickhami*; USA, Baja California, Mexico)^1,18,20–23^, and three species from the Southwestern Pacific Ocean (*C. australiensis, C. tasmanica,* and *C. humesi*; Australian coast)^21,24^. Not a single representative of the family Carcinonemertidae has been described so far from the South-eastern Pacific Ocean (SEP).

In the SEP, several species of brachyuran crabs, most of them belonging to the family Cancridae, are of commercial value^25,26^. In the Chilean coast, eight species with a wide distribution along the continental shelf are targeted by artisan fisheries: *Cancer edwardsi*, *C. coronatus*, *C. porteri*, *Homalaspis plana*, *Ovalipes trimaculatus*, *Taliepus marginatus*, *T. dentatus,* and *Romaleon setosum* (previously, *C. setosus)*. Importantly, *R. setosum* and *C. porteri* represent two of the most heavily targeted species by local fisheries along the coast of Chile. *Cancer porteri* is distributed from Panama (9° S) to Concepción Bay (36° S), Chile^27^, and is commonly extracted by artisan fishermen in central Chile (32°–38° S). In turn, *R. setosum* is distributed from the coast of Ecuador (5° S) to Peninsula de Taitao (47° S), Chile and is extracted between 24° S and 40° S^28,29^.

The capture of gravid female crabs of both *R. setosum* and *C. porteri* is prohibited year-round, these fisheries are closed during the reproductive season, and a minimum catch body size (12 mm carapace width) has been established by the Fisheries and Aquaculture Sub-Secretary of the government of Chile since 1991^30,31^. Importantly, during the last six years, crab landings (tons) have decreased significantly in Chile^25^. For instance, *C. porteri* landings decreased from 667 tons in 2014 to 2.62 tons in 2019. In turn, landings diminished from 548 to 216 tons in the same period for *R. setosum*^32^. This steady temporal decrease in landed biomass has forced the implementation of specific management strategies for the two crab species, including a full fishery ban in the case of *C. porteri*^28^.

Despite the importance of *R. setosum* and *C. porteri* along the Chilean coast, several biological aspects of these crabs are not known, and the presence of nemertean egg-predators has not been previously studied. Nemertean egg-predators, if present, could affect the reproductive performance and population dynamics of these crabs and result in the collapse of their fisheries, as it has been reported before in the north Pacific coast^12^. Consequently, the objective of this study was to explore, for the first time, the presence and prevalence of nemertean egg-predators in the commercial crabs *Cancer porteri* and *Romaleon setosum*, in the central and northern coast of Chile (SEP). Importantly, detailed morphological and molecular analyses of the nemertean worms infecting the aforementioned crabs revealed that they belong to a single species of *Carcinonemertes* not yet described. Here, we formally describe this new species as *Carcinonemertes camanchaco* sp. nov.

## Results

### Prevalence of nemerteans per host species and locality

**Figure 1:**
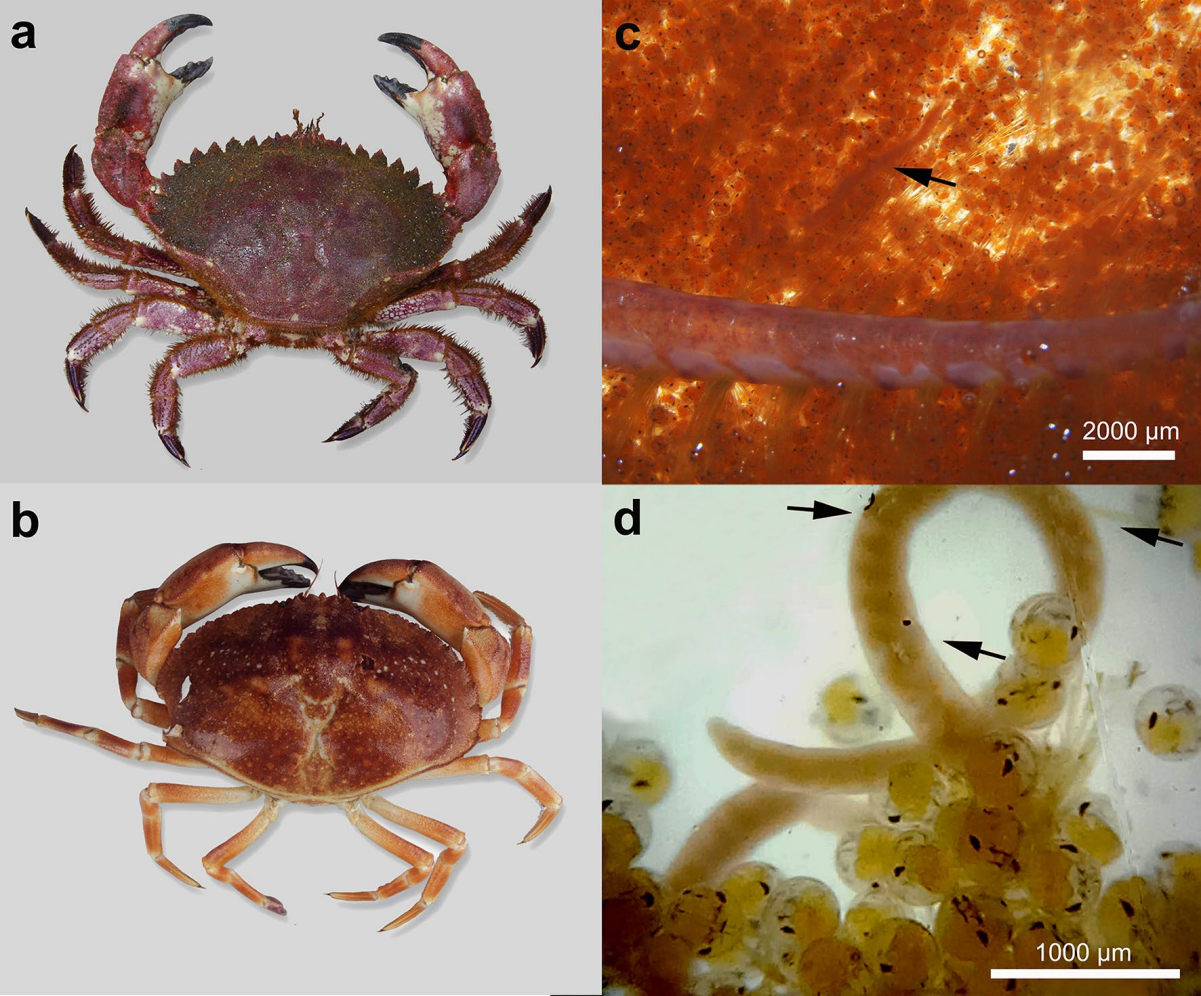
Host crab species *Romaleon setosum* (**a**) and *Cancer porteri* (**b**) infected by *Carcinonemertes camanchaco* sp. nov*.* present in their egg masses (**c**, **d**).

A total of 48 female crabs (non-ovigerous) of *Cancer porteri* from Iquique (20° S), 50 gravid females of *Romaleon setosum* from Coquimbo (30° S), and 21 gravid females of *C. porteri* from Valparaíso (33° S) were examined. Nemerteans were only detected in egg masses of gravid female crabs (Fig. 1); therefore, the prevalence of nemerteans in specimens from Iquique was 0%. The prevalence of nemerteans in *C. porteri* and *R. setosum* from Coquimbo and Valparaíso was 24.0% and 38.1%, respectively. We found several nemertean developmental stages (eggs, juveniles, and adult worms) infecting the egg masses of the infected crabs. Juvenile worms were found either roaming among the crab eggs or encapsulated on the surface of the crab’s abdomen. Adult worms were found only in egg masses, covered by a mucus sheath (not ornamented). Long strands of worm eggs covered by translucent mucus were also observed among the eggs of ‘berried’ (= brooding) female crabs.

### Morphometric analyses

The studied male worms had a mean ± SD body size of 3.64 ± 0.59 mm and the female worms had a mean body size of 5.84 ± 1.25 mm. A t-test demonstrated statistically significant differences in average body size between male and female worms (t = 4.42, d.f. = 19, P < 0.01), with the females being larger than the males. For both male and female worms, body length was not correlated with stylet length, basis length, and stylet-basis ratio (Spearman correlations; all P > 0.05).

**Figure 2:**
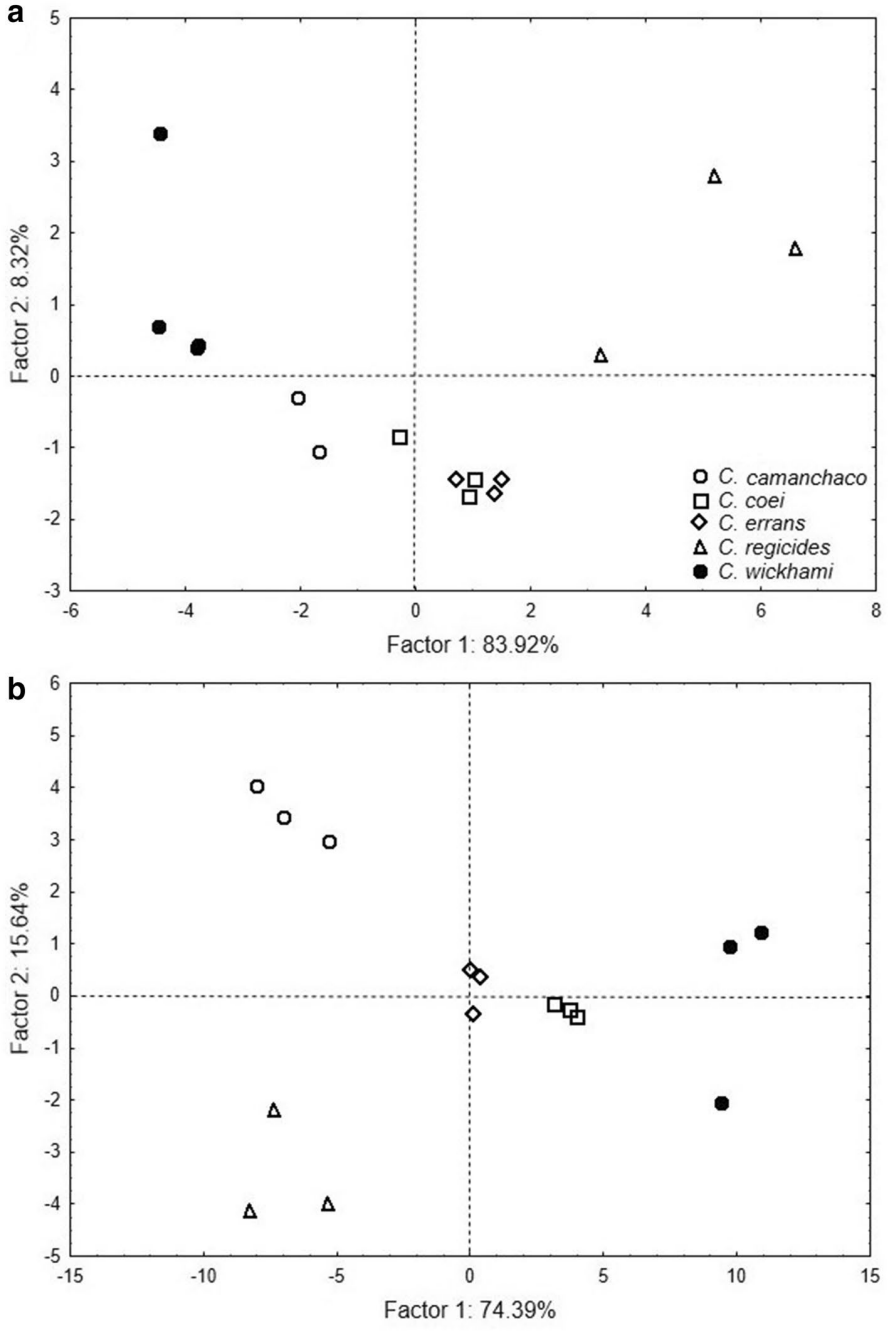
Principal components analysis based on morphometric measurements standardized by total length from five species of *Carcinonemertes*. (**a**) Female worms, (**b**) Male worms. White circles = *C. camanchaco* sp. nov., square = *C. coei*, diamond = *C. errans*, triangle = *C. regicides*, black circle = *C. wickhami*.

The PCAs performed separately for female and male worms infecting the studied crabs in the SEP and those from other four closely related species of *Carcinonemertes,* characterized by having two gonad rows on each side of the intestine, indicated that > 95% of the variability in body proportions among the studied specimens and species was explained by the first three PCA axes (Table 1). EW/BL, DW/BL, BW/BL, and SL/SBL/BL (factor loads > 0.95; Table 1) were the most important measures that distinguished both male and female nemertean worms collected during this study from specimens belonging to the other four similar, previously described species (Fig. 2a, b). The DFA, using the orthogonal components of the PCA, revealed significant differences among female (Wilks’ Lambda = 0.01; F (12, 21) = 7.62; p < 0.01) and male (Wilks’ Lambda = 0.001; F (12, 21) = 24.96; p < 0.01) nemertean specimens of the four species. Additionally, the DA showed that 93% of the female specimens and 100% of the male specimens were correctly assigned to their respective nemertean species.

**Table 1:** Factor loadings of the PCA using morphometric data obtained from females and males of *Carcinonemertes camanchaco* sp. nov., based on a correlation matrix.

	Female		Male	
	Factor 1	Factor 2	Factor 1	Factor 2
SL/BL	− 0.850825	0.360003	− 0.794413	0.412755
BW/BL	− 0.707444	− 0.682767	− 0.953003	0.163532
EL/BL	− 0.956083	− 0.063183	− 0.483576	− 0.845918
EW/BL	− 0.935800	− 0.130158	− 0.979213	0.122925
MCW/BL	− 0.980137	0.095331	− 0.804470	0.286164
PPCL/BL	− 0.883926	0.184063	− 0.827251	− 0.482428
SBL/BL	− 0.992018	0.067382	− 0.980231	− 0.008689
SL/SBL/BL	− 0.986408	0.036445	− 0.962674	− 0.018336

Measurements: BL = total body length, BW = body width, EL = eye length, EW = eye width, MCW = middle chamber width, SL = stylet length, SBL = stylet basis length, PPCL = posterior proboscis chamber length.

### Phylogenetic analyses

The phylogenetic analyses included 21 *cox1* sequences 620 bp in length (after alignment): 8 sequences obtained during this study and 13 sequences retrieved from GenBank. The data matrix comprised a total of 205 parsimony informative sites.

**Figure 3:**
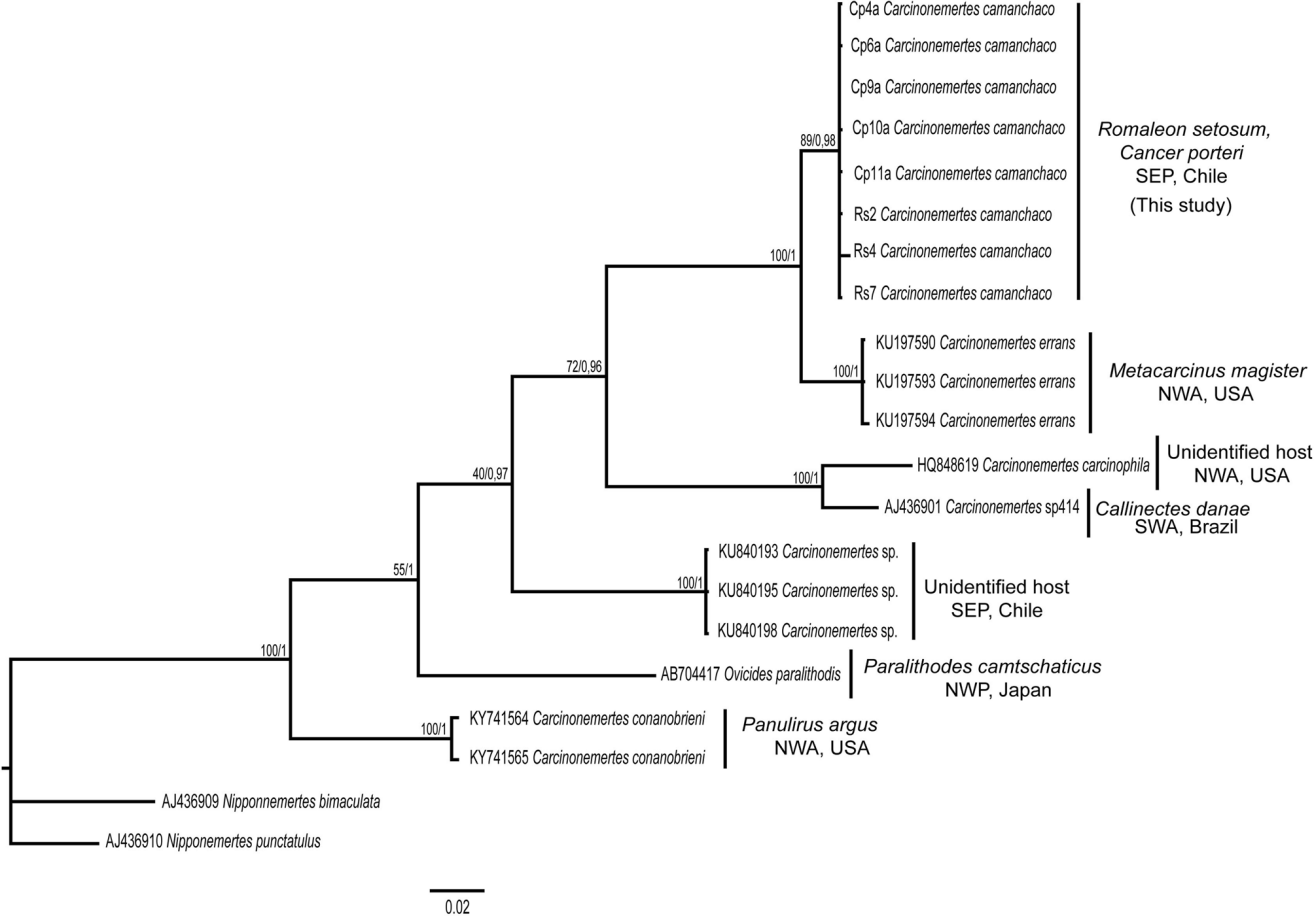
Phylogenetic tree based on the *cox1* gene for *Carcinonemertes* spp. from different host species, inferred using Bayesian inference (BI) and Maximum Likelihood (ML) analyses. Numbers near internal nodes denote posterior probabilities or bootstrap values from the BI and ML phylogenetic analyses (BI/ML), respectively. Sequences for *Nipponnemertes bimaculata* and *N. punctatulus* were used as outgroups. *SEP* South eastern Pacific, *NWA* North western Pacific, *SWA* South western Atlantic, *NWP* North western Pacific.

ML and BI analyses (bootstrap support of 89 and 1.0 for ML and BI, respectively), demonstrated that the specimens of *Carcinonemertes* infesting *R. setosum* (three sequences) and *C. porteri* (five sequences) belong to the same species. Furthermore, both ML and BI phylogenetic reconstructions indicated that the studied specimens of *Carcinonemertes* form a clade sister to *C. errans,* a worm that infects *Metacarcinus magister* in the coast of California (Fig. 3). The intra-specific genetic distances calculated for the 8 sequences of this study varied between 0 and 0.3% (Table 2). The mean genetic distance between specimens of *Carcinonemertes* collected for this study and other species belonging to the genus *Carcinonemertes* was 3.6% (3.6–4.2%, n = 3 sequences) versus *C. errans* (*ex Metacarcinus magister*), 17% (16.8–17.2%, n = 3 sequences) versus *Carcinonemertes* sp*.* from an unknown host species previously collected from Chile, and 23.6% (23.6–23.7%, n = 2 sequences) versus *C. conanobrieni* (*ex Panulirus argus*) (Table 2). The phylogenetic trees also indicated that the genus *Carcinonemertes* is paraphyletic, given the inclusion of *Ovicides* in the same well-supported phylogenetic clade containing all species of *Carcinonemertes* (Fig. 3).

**Table 2:** Genetic distance between sequences of *Carcinonemertes* spp. available in GenBank and sequences of *C. camanchaco* sp. nov. provided in this study.

	Species	1	2	3	4	5	6	7	8	9
1	*Nipponnemertes bimaculata* (n = 1)									
2	*Nipponemertes punctatulus* (n = 1)	0.086	0							
3	*Ovicides paralithodis* (n = 1)	0.289	0.282	0						
4	*Carcinonemetes errans* (n = 3)	0.300	0.292	0.218	**0.001**					
5	*Carcinonemertes conanobrieni* (n = 2)	0.219	0.212	0.201	0.239	**0.003**				
6	*Carcinonemertes* sp (n = 3)	0.245	0.247	0.197	0.205	0.209	**0**			
7	*Carcinonemertes carcinophila* (n = 1)	0.250	0.230	0.218	0.219	0.235	0.194	0		
8	*Carcinonemertes* sp414 (n = 1)	0.228	0.216	0.229	0.184	0.231	0.200	0.055	0	
9	*Carcinonemertes camanchaco* (n = 8)	0.282	0.271	0.219	0.036	0.236	0.168	0.209	0.180	**0.001**

In bold, intra-specific distances (sequences > 2).

### Morphological characterization of specimens from the SEP

We described morphological characteristics including body color and body measurements of female and male nemertean worms. Comparisons of morphology, morphometry, host species and geographical distributions of *Carcinonemertes camanchaco* sp. nov. and the other 16 *Carcinonemertes* species described around the world are presented in Supplementary Table S1. Detailed morphological description for *Carcinonemertes* spp. is provided in the Taxonomy section.

### **TAXONOMY: *Carcinonemertes camanchaco***

**Figure 4:**
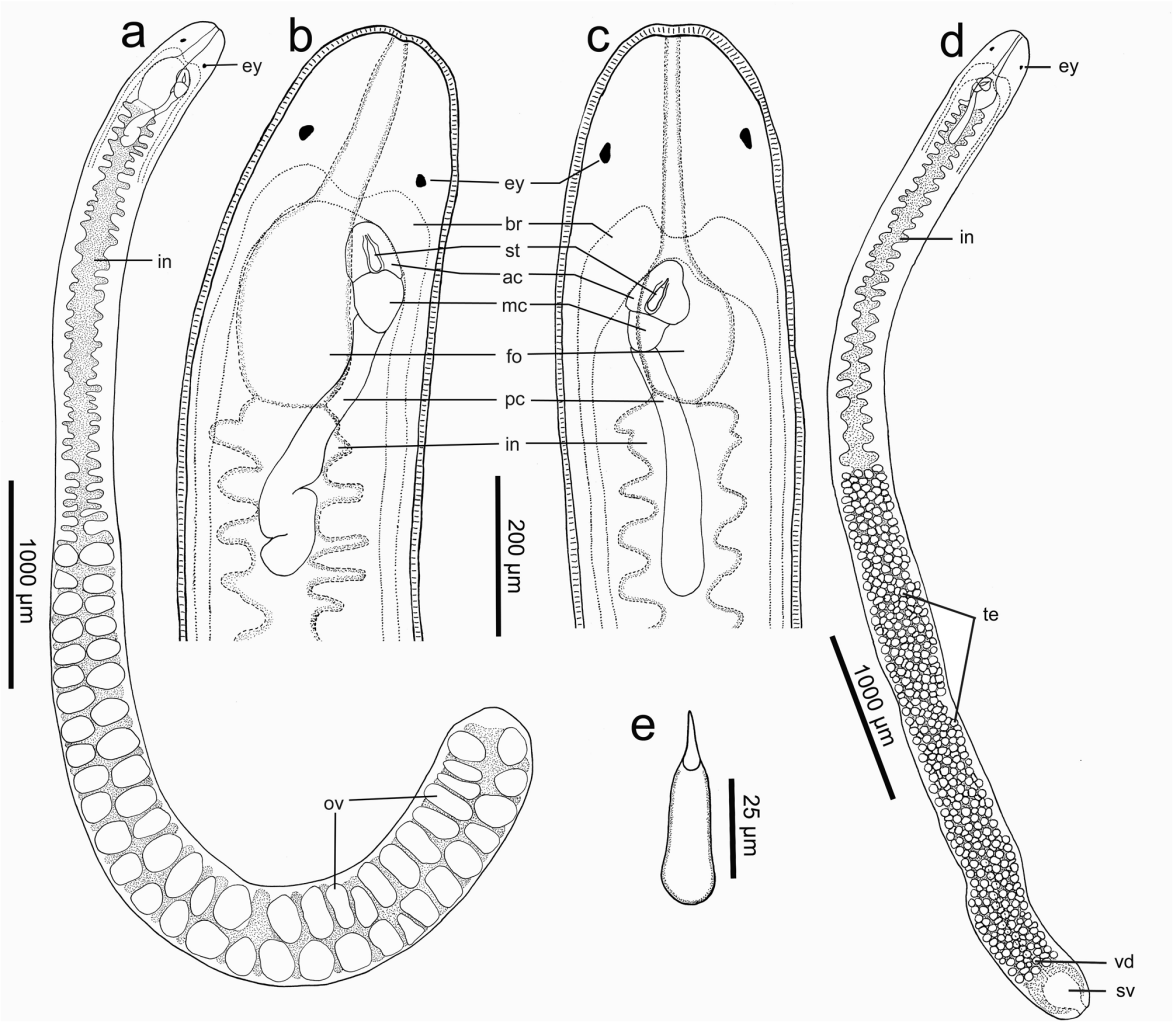
Adult *Carcinonemertes camanchaco* sp. nov. (**a**) Complete female worm, (**b**) anterior part of a female worm, (**c**) anterior part of a male worm, (**d**) complete male worm and (**e**) Stylet. *ey* eyes, *br* brain lobe, *st* stylet, *ac* anterior chamber, *mc* medium chamber, *fo* foregut, *pc* proboscis chamber, *in* intestine, *ov* ovary, *te* testes, *sv* seminal vesicle.

**Figure 5:**
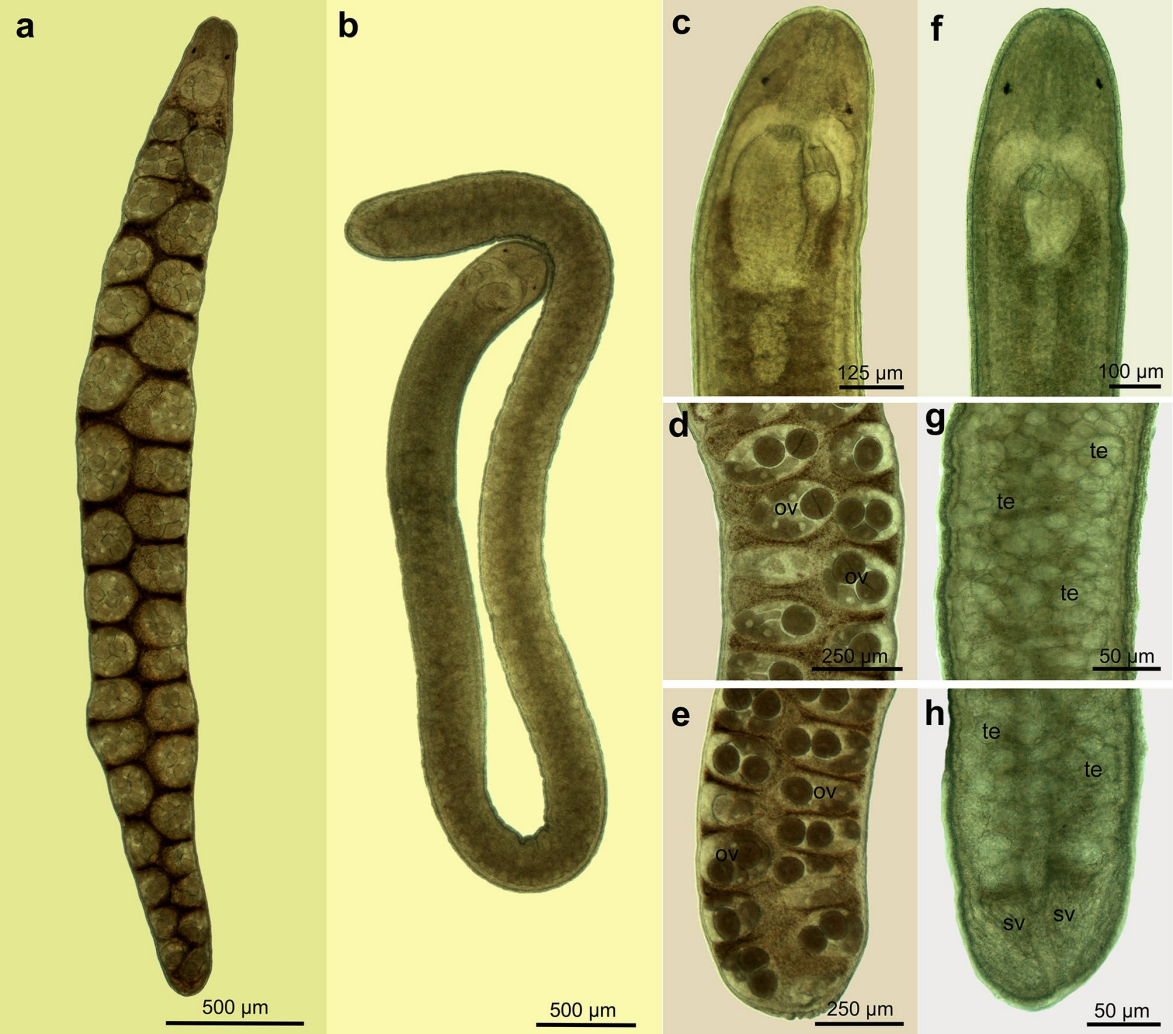
Photograph of complete worm of *Carcinonemertes camanchaco* sp. nov. (**a**) Complete female worm, (**b**) complete male worm; (**c**) anterior; (**d**) trunk and (**e**) posterior female body; (**f**) anterior; (**g**) trunk and (**h**) posterior male body. Body organs nomenclature is given in Fig. 4.

**Figure 6:**
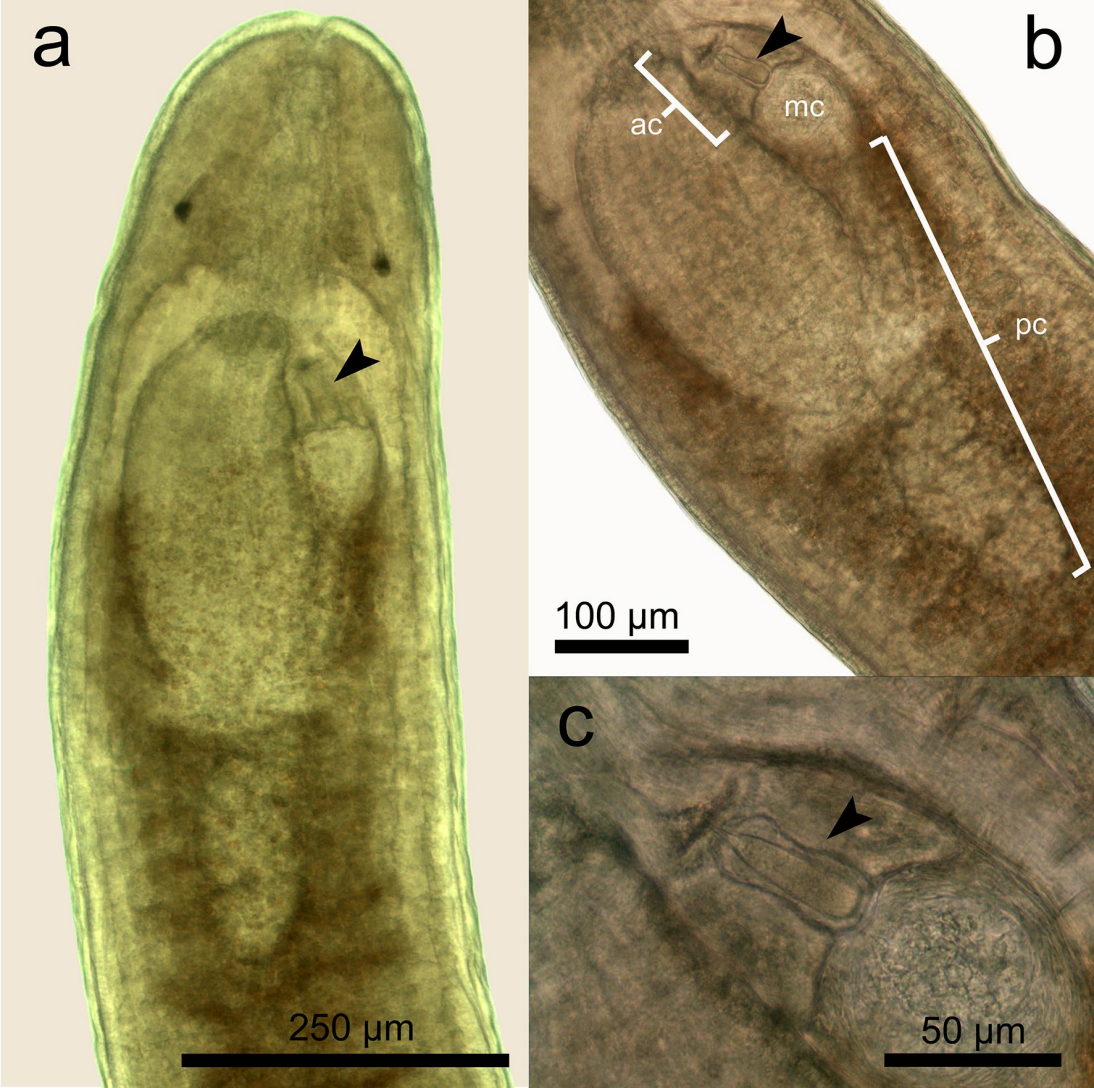
Photographs of anterior body section of *Carcinonemertes camanchaco* sp. nov (**a**); showing details of proboscis chamber, median chamber, and anterior chamber (**b**) and stylet (**c**).

 N.V. Leiva, L. Ñacari., J.A. Baeza, & M.T. Gonzalez, sp. nov. (Figs. 4, 5 and 6). ZooBank registration: To comply with the regulations set out in article 8.5 of the amended 2012 version of the International Code of Zoological Nomenclature (ICZN), details of the new species have been submitted to ZooBank. The Life Science Identifier (LSID) of the article is urn:lsid:zoobank.org:pub:DCF78A3D-19E6-407F-9ED9-47DADAF2521A. The LSID for the new name *Carcinonemertes camanchaco* is urn:lsid:zoobank.org:act:0DCA7DF8-1E8D-4AF5-A3E9-141BEC061BDD.

Phylum Nemertea.

Class Hoplonemertea Hubrecht, 1879.

Family Carcinonemertidae Sumner, Osburn & Cole, 1913.

Genus *Carcinonemertes* Coe, 1902.

**Etymology** The specific name refers to the 'Camanchaco’ ethnic group, indigenous people that inhabited the north and central coast of Chile and whose descendants are legally recognized as original indigenous people since 2020 in Chile.

**Site of infection/host** Adult specimens were found in the egg masses of hosts *Cancer porteri* (Rathbun, 1930) and *Romaleon setosum* (Molina, 1782) (Crustacea, Decapoda).

**Prevalence and mean intensity (± SD)** 24.0% and 2.6 (± 2.07) in *Cancer porteri* (Valparaiso) and 38.1% and 3.8 (± 2.4) in *Romaleon setosum* (Coquimbo).

**Host habitat and sampling locality** Subtidal, Puerto Aldea, Coquimbo (30°17´37´´S, 71°36´36´´W) and Caleta Portales, Valparaíso (33°1′52.74″S, 71°35′25.18″W), north and central Chilean coast, collected during November 2020.

**Materials examined** Holotype (one female): MNHNCL NEME-15002; paratype (one male): MNHNCL NEME-15003; paratype (three males): MNHNCL NEME-15004, and paratype (three females): MNHNCL NEME-15005.

**Description** Body color varies from translucent to pale orange due to gonads and internal organs’ coloration. The anterior and posterior end of the body is rounded (Fig. 4). Worms are filiform in shape and range from 2.381 mm to 8.922 mm in total length. The anterior proboscis chamber could not be distinguished. The middle chamber contains a single stylet (Figs. 5, 6). The stylet appears like a dagger with a broad- stylet basis (Figs. 4e, 5 and 6). Accessory stylets are not present. Posterior proboscis chamber is elongated. Foregut (anterior portion of the esophagus) joins the rhynchodaeum at or just anterior to the brain (Figs. 4, 5). Stomach opens into the intestine, which presents numerous paired unbranched diverticula (Figs. 4, 5). The anus is located at the terminal posterior portion of the body. All body measurements are given in Table 3.

**Table 3:** Measurements of *Carcinonertes camanchaco* sp. nov.

Character	Female (n = 18)			Male (n = 9)		
	Mean	SD	Range	Mean	SD	Range
Body length	5.837	1.254	4.286–8.922	3.643	0.857	2.381–4.929
Body width	0.313	0.121	0.143–0.574	0.222	0.048	0.143–0.286
Eye length	0.030	0.009	0.020–0.049	0.021	0.006	0.014–0.030
Eye width	0.019	0.006	0.009–0.030	0.018	0.009	0.007–0.030
Distance: between eyes	0.099	0.030	0.050–0.163	0.101	0.028	0.070–0.162
Distance: eyes to tip of head	0.120	0.028	0.080–0.177	0.101	0.014	0.090–0.133
Brain lobe length	0.186	0.047	0.109–0.247	0.154	0.028	0.117–0.200
Brain lobe width	0.049	0.008	0.040–0.060	0.047	0.014	0.026–0.075
Distance: brain to tip of head	0.176	0.030	0.120–0.229	0.162	0.037	0.102–0.222
Diaphragm length	0.069	0.014	0.051–0.100	0.067	0.015	0.048–0.100
Diaphragm width	0.052	0.009	0.043–0.061	0.057	0.011	0.045–0.080
Proboscis bulb length	0.044	0.010	0.025–0.060	0.042	0.016	0.026–0.075
Proboscis bulb width	0.043	0.003	0.039–0.048	0.040	0.012	0.030–0.061
Posterior proboscis chamber length	0.143	0.056	0.090–0.260	0.147	0.061	0.100–0.256
Posterior proboscis chamber width	0.069	0.010	0.055–0.080	0.048	0.004	0.042–0.052
Single stylet length	0.009	0.001	0.006–0.010	0.013	0.006	0.006–0.020
Single stylet width	0.004	0.001	0.003–0.005	0.005	0.001	0.005–0.006
Stylet basis length	0.041	0.010	0.026–0.060	0.029	0.005	0.020–0.037
Stylet basis width	0.010	0.002	0.008–0.014	0.015	0.004	0.010–0.020
Stylet: basis ratio	0.235	0.042	0.33333333	0.462	0.241	0.167–0.800
Distance: stylet to tip of head	0.263	0.048	0.167–0.331	0.243	0.061	0.183–0.305
Number of ovaries	71.786	24.931	40–126		–	
Seminal vesicle length		–		0.183	0.040	0.125–0.240*
Distance: first gonad to tip of head	1.074	0.437	0.341–2.016	1.129	0.304	0.693–1.710

All measurements are given in mm (exceptions include: Stylet: basis ratio, and the number of ovaries [direct count]).

*SD* standard deviation, *Two specimens analyzed.

**Females (18 living specimens)** Dimensions of relaxed worms were 5.837 ± 1.254 (mean ± SD, range = 4.880–8.922) long and 0.313 ± 0.121 (0.167–0.574) wide. Two irregular black eyes, 0.030 ± 0.009 (0.020–0.049) long and 0.019 ± 0.006 wide (0.009–0.030) (Fig. 4). Distance between eyes 0.099 ± 0.030 (0.050–0.163 mm). Distance from eyes to tip of head 0.120 ± 0.028 (0.080–0.177). The brain is 0.186 ± 0.047 (0.109–0.247) long and 0.049 ± 0.008 (0.040–0.060) wide (Fig. 4b, 4c). Distance from brain to tip of head 0.176 ± 0.030 (0.155–0.229). Middle chamber is 0.069 ± 0.014 (0.051–0.100) long and 0.052 ± 0.009 (0.043–0.06) wide (Figs. 4b, 6). Stylet 0.009 ± 0.001 (0.006–0.010) long. Stylet basis 0.041 ± 0.010 (0.026–0.060) long and 0.010 ± 0.002 (0.008–0.014) wide (Figs. 4e; 6). Stylet: basis ratio 0.235 ± 0.042 (0.167–0.33). Distance from central stylet to tips head 0.263 ± 0.048 (0.183–0.331). Proboscis bulb 0.044 ± 0.010 (0.025–0.060) long and 0.043 ± 0.003 (0.039–0.048) wide. Posterior proboscis chamber is elongated, 0.143 ± 0.056 (0.099–0.260) long and 0.069 ± 0.010 (0.055–0.080, n = 6) wide (Figs. 4b, 6). Ovaries are orange and numerous (n = 72 ± 25 [range: 40–126] per worm); regularly distributed in two rows on each side of the intestinal diverticule (Figs. 4a, 5a, d, e). Distance from first gonad to tip of head 1.074 ± 0.437 (0.341–2.016).

**Males (9 living specimens)** Dimensions of relaxed worms were 3.643 ± 0.857 (2.381–4.929) long and 0.222 ± 0.048 (0.143–0.280) wide. Two irregular black eyes, 0.021 ± 0.006 (0.014–0.030) long and 0.018 ± 0.009 wide (0.008–0.030) (Fig. 4c, d). Distance between eyes 0.101 ± 0.028 (0.070–0.162). Distance from eyes to tip of head 0.101 ± 0.014 (0.090–0.133). Brain is 0.154 ± 0.028 (0.117–0.200) long and 0.047 ± 0.014 (0.026–0.075) wide (Fig. 4c). Distance from brain lobe to tip of head 0.162 ± 0.037 (0.102–0.222). Middle chamber is 0.067 ± 0.015 (0.048–0.100 mm) long and 0.057 ± 0.011 (0.045–0.080) wide (Figs. 4c, 6). Single stylet on basis, 0.011 ± 0.004 (0.006–0.020) long (Figs. 4e, 6). Stylet basis 0.030 ± 0.006 (0.020–0.037) long and 0.013 ± 0.003 (0.010–0.015) wide. Stylet: basis ratio 0.366 ± 0.170 (0.167–0.667). Distance from central stylet to tips head 0.243 ± 0.061 (0.183–0.305). Proboscis bulb 0.042 ± 0.016 (0.026–0.075) long and 0.040 ± 0.012 (0.030–0.061) wide (Figs. 4c, 6). Posterior proboscis chamber elongated 0.147 ± 0.061 (0.100–0.256) long and 0.048 ± 0.004 (0.042–0.052) wide (Figs. 4c, 6). Testes are numerous and translucent, occupying all the middle and rear section of body, between intestinal diverticula. A single seminal vesicle was observed near the posterior end, 0.183 ± 0.040 (0.125–0.240, n = 2) (Table 3; Fig. 6).

**Differential diagnosis** *Carcinonemertes camanchaco* sp. nov. closely resembles *C. errans*, *C. coei*, *C. wickhami*, and *C. regicides* due to the presence of two gonad rows on each side of the intestine. The other 12 described species of *Carcinonemertes* have only a single gonad row (Table S1). *Carcinonemertes camanchaco* sp. nov. can be distinguished from *C. errans* due to the presence of a mucus sheath, present in *C. camanchaco* sp. nov. but absent in *C. errans. Carcinonemertes wickhami* has been reported to build mucus sheaths as here reported for *C. camanchaco* sp. nov. However, in *C. camanchaco* sp. nov. the mucus sheath is simple, while in *C. wickhami* the mucus sheath is ornamented. *C. camanchaco* sp. nov. differs from *C. coei* in body colour, described as translucent white to pale orange in *C. camanchaco* sp. nov. and yellowish-white in *C. coei*. The ratio of the stylet/basis is smaller in *C. camanchaco* sp. nov. (0.233) than in *C. errans* (0.321)*, C. coei* (0.381), *C. wickhami* (0.528) and *C. regicides* (0.432). A summary of the morphological and morphometric characteristics of the different species of *Carcinonemertes* are given in Supplementary Table S1.

## Discussion

This is the first study that has tested for the presence/absence of nemertean egg-predators in commercial crabs from the SEP coast. We did find nemertean worms infecting two species of crabs from the SEP and our morphometric and molecular analyses (based on the mitochondrial *cox*1 gene) indicated that these nemerteans parasitizing eggs of *Romaleon setosum* and *Cancer porteri* belong to the same species of *Carcinonemertes*, here named *C. camanchaco* sp. nov. The same analyses also indicate that this newly discovered entity is genetically different from all other known species of *Carcinonemertes* with information available in GenBank. We note that the *cox1* is not useful to distinguish among species in some clades within the Nemertea (Sundberg et al., 2016). An integrative taxonomic approach, including the (classical) comparison of morphological traits, multivariate morphometric analyses, and molecular data (*cox1* mitochondrial gene) demonstrated that the nemertean inhabiting two sympatric crab species is a new species. The nemertean species recorded in our study is characterized by the presence of two ovaries on each side of the intestine and the production of a simple mucus sheath (not ornamented). The combination of these morphological characters plus genetic characters clearly distinguished our specimens from previously described species in the same genus.

The genus *Carcinonemertes* now comprises 17 valid species. However, genetic information (i.e., *cox1*) exist for only five species (plus the new species), which were used here to construct a phylogenetic tree. This tree showed that *C. conanobrieni*, parasitizing Caribbean spiny lobsters, is sister to a clade comprised of congeneric species infecting brachyuran crabs (Fig. 3). Only two nemertean species have been reported before from crabs belonging to the family Cancridae: *C. errans* described from *Metacarcinus magister*^33^ and *C. camanchaco* sp. nov. from *Cancer porteri* and *Romaleon setosum* (this study). The phylogenetic tree indicated that *C. camanchaco* sp. nov. and *C. errans* are sister species (with 3.6% of genetic distance). These nemertean species parasitizing two phylogenetically close host species, belonging to the family Cancridae, implies that the genus *Carcinonemertes* could be coevolving together with different species used as hosts^6,14^.

Estimating the prevalence of nemertean egg-predators is exceedingly relevant considering the impact that *Carcinonemertes* and *Ovicides* have on the reproductive performance and population dynamics of their host species^34,35^. Notably, studies reporting the prevalence of nemerteans are rare. Santos et al.^13^ found that the prevalence of *C. divae* in female and male crabs of *Libinia spinosa* was 87% and 43.2%, respectively. In turn, *C. errans* prevalence in *Metacarcinus magister* is remarkably high; up to 97% of the gravid females can be infected by this nemertean during one year/ fishing season. Importantly, the intensity of *C. errans* on infected *Metacarcinus magister* can also be very high^5^. In the 70’s, *C. errans* became an epidemic, resulting in the direct mortality of an average of 55% of the eggs produced by females of *M. magister*. The above suggests that *C. errans* might probably be one of the most numerically significant predators on these crabs and was responsible for the collapse of this fishery in Central California^5,12,33^. The prevalence of *C. camanchaco* sp. nov. was lower than that reported in the aforementioned species (38% in *R. setosum* and 24% in *C. porteri*). Given the previously demonstrated impact of nemerteans on the fecundity of their respective host species, and the considerable and steady decreases in landings reported for *R. setosum* and *C. porteri* along the coast of Chile in the last four years, we argue that additional studies are needed to evaluate the effect of *C. camanchaco* sp. nov. on the fertility and population dynamics of these crabs.

The prevalence of several parasite species can vary across short- and long-time scales and can be associated to environmental variability and/or host availability^11^. Shields and Kuris^3^ showed that prevalence of *C. epialti* on the shore crab *Hemigrapsus oregonensis* varied between years, finding that during non-outbreak periods prevalence was 48%, while during the outbreak periods this prevalence increased to 97%. Campbell et al.^24^ showed that prevalence of *C. australiensis* was 67% in females of the spiny lobster *Panulirus cygnus*. Most recently, Baeza et al.^35^ determined that the prevalence of *C. conanobrieni* in the lobster *Panulirus argus* was 7.4%, and Simpson et al.^10^ showed that prevalence increased to 93.9% a year later (107 of 114 gravid females). This information suggests that nemertean infection levels can change quickly between consecutive years. Therefore, we argue in favor of additional studies to determine short- and long-term spatial and temporal variability in the prevalence and intensity of *C. camanchaco* sp. nov. in the different species infected, as well as the effect of this parasite on the reproductive performance and overall health of its crab hosts. Carrying out temporal monitoring of the newly discovered nemertean species across host species is needed to manage these heavily fished species towards the goal of sustainability in Chile.

## Methods

### Sampling and parasite collection

Females of *Cancer porteri* and *Romaleon setosum* were collected by divers from three localities, Caleta Chanavayita, Iquique (20°42′10″S, 70°11′15″W), Puerto Aldea, Coquimbo (30°17´37´´S, 71°36´36´´W), and Caleta Portales, Valparaíso (33°1′52.74″S, 71°35′25.18″W). We collected a total of 48 crabs belonging to *Cancer porteri* from Iquique, 50 crabs belonging to *Romaleon setosum* from Coquimbo, and 21 specimens of *C. porteri* from Valparaíso. The samples of *C. porteri* collected in Iquique and Valparaíso were transported to the laboratory at the Universidad de Antofagasta, and alive specimens of *R. setosum* were transported to the laboratory at the Universidad Católica del Norte, Chile. Collection of these specimens was authorized by the Fishery and Aquaculture Sub-Secretary of the government of Chile (R. Ex N° E-2020–481).

In the laboratory, each crab (either alive or frozen [− 10 °C]) was examined for the presence or absence of nemertean worms. All eight pleopods were removed from each studied crab, and all embryos were gently stripped away from the pleopods of each gravid female using fine forceps and placed into Petri dishes with filtered seawater (10 µm). The egg mass of each gravid female, as well as the abdomen, pleopods, gills, and branchial chambers from all analyzed crabs were inspected under a stereomicroscope (Leica M80, Leica Microsystems, Wetzlar, Germany) to determinate the presence or absence of nemertean worms. Nemerteans collected from gravid female crabs were placed in Petri dishes with filtered seawater up to the point in time when measurements and photographs were taken, and morphological characters were studied in detail. Nemerteans were first relaxed in a 1:1 solution of 1 M MgCl_2_ (prepared with distilled water) and seawater for 1–5 min. The specimens were fixed in formaldehyde at 10% for morphological examination and 99% EtOH solution for genetic analyses.

### Morphometric study and morphological characterization

Different body parts were measured in all adult worms (in mm, precision = 0.01 mm) using a digital camera (Mshot MD90) connected to a stereomicroscope (Olympus SZX7, Tokyo, Japan) and the programs MShot Image Analysis System 1.5.2 and ImageJ^36^. We measured total body length (BL), body width (BW), ocelli length (OL), ocelli width (OW), diaphragm width (DW), stylet length (SL), and stylet basis length (SBL) of each studied specimen. The different body measurements of the studied specimens were compared to those from all other previously described species of *Carcinonemertes* (n = 16).

Principal component analyses (PCAs) were also performed separately for both male and female worms, using body part measurements standardized by BL. For these analyses, only previously described nemertean species with taxonomic characteristics most similar to the specimens collected during this study were used; only species that exhibit two rows of gonads (i.e., *Carcinonemertes coei, C. errans, C. regicides, C. wickhami*). The body proportions used were BW/BL, OL/BL, OW/BL, DW/BL, SL/BL, SL/SBL, SBL/BL and SL/SBL/BL. The first two axes of the PCA were also used to evaluate the importance of each measured ratio on the morphometric variability observed among different species of *Carcinonemertes*. Finally, orthogonal variables (principal components), which preserve the original information of the database, were used to perform a Discriminant Function Analysis (DFA) and to assign specimens to the different worm species^37,38^. All statistical analyses were conducted using the software Statistica 10.0 (Statsoft, Tulsa, USA).

### DNA isolation, PCR amplification and sequencing

A total of eight juvenile worms were dissected with forceps and DNA was isolated from each one of them separately using a protocol modified from Miller et al.^39^ that included treatment of each specimen with sodium dodecyl sulphate and digestion with Proteinase K. Proteins were removed by precipitation with NaCl, and the DNA was finally precipitated with ethanol. The resultant DNA was eluted in nuclease-free water and quantified using a spectrophotometer until a concentration of 30 to 50 ng/μL was reached.

The mitochondrial gene cytochrome c oxidase 1 (*cox1*) was amplified using universal primers described in Folmer et al.^40^. Each PCR reaction had a final volume of 35 μl including: 0.175 U/μl Taq polymerase (GoTaq G2, Promega), 7 μl 5X PCR buffer, 5.6 μl MgCl_2_ (25 mM), 2.1 μl bovine serum albumin (10 mg/ml), 0.7 μl of deoxynucleotide triphosphate (dNTP; 10 mM), 1.4 ul 10 pM each primer, and 3 μl template DNA. PCR amplifications were performed in a Boeco Ecogermany M-240R thermal cycler using the following optimal cycling conditions: 95 °C (5 min), followed by 40 cycles at 95 °C (45 s), 50 °C (45 s) and 72 °C (1 min), and a final extension step at 72 °C (10 min). PCR products were sent to Macrogen (Seoul, Korea; http://www.macrogen.com) for purification and sequencing of both forward and reverse DNA strands.

Sequences were edited and contigs were assembled using ProSeq 2.9 beta^41^. All haplotype sequences obtained during this study were deposited in GenBank under the following accession numbers MW596479-MW596486.

### Phylogenetic analyses

Four other species of *Carcinonemertes* were used as ingroup terminals for molecular comparisons with our new species, and two species of *Nipponnemertes* were included in the analysis as outgroup terminals. All sequences were first aligned using the software Clustal W^42^. The aligned dataset was analyzed with the software JModelTest2^43^ which compares different models of DNA substitution in a hierarchical hypothesis-testing framework to select a base substitution model that best fits the data for each gene. The best model found by JModelTest2, selected with the corrected Akaike information criterion^44^, was GTR + I. The model parameters were as follows: assumed nucleotide frequencies A = 0.1988, C = 0.1266, G = 0.2261, and T = 0.4485; substitution rate matrix with A-C substitution = 0.0025, A-G = 11.4049, A-T = 2.8337, C-G = 0.1128, C-T = 7.4120, G-T = 1.000, and p-inv distribution with shape parameter 0.6420. Next, the best model was implemented in MrBayes 3.2.7a^45^ for Bayesian Inference analysis (BI) and in IQ-TREE^46^ for Maximum Likelihood analysis (ML). All phylogenetic analyses were conducted in the CIPRES Science Gateway V. 3.3 platform (http://www.phylo.org/)^47^.

For the BI analysis, unique random starting trees were used in the Metropolis-coupled MCMC^45^. The analysis was performed for a total of 5,000,000 generations. Visual inspection of log-likelihood scores against generation time indicated that the log-likelihood values reached a stable equilibrium before the 100,000th generations. Thus, a burn-in of 1000 samples was conducted; every 100th tree was sampled from the MCMC analysis, obtaining a total of 100,000 trees, and a consensus tree with the 50% majority rule was calculated for the last 59,000 sampled trees. Support for nodes in the BI tree topology was obtained by posterior probability. For the ML analysis, we used the default options in IQ-TREE run through the Cypress Science Gateway^47^. The robustness of the ML tree topology was assessed by bootstrap iterations of the observed data 1000 times. Phylogenetic trees were visualized and edited in Figtree 1.4.4.

Pairwise genetic distances (intra- and inter-specific) between the sequences of *cox1* gene were calculated in MEGA 6^48^ using the Kimura 2-Parameter model^49^.

## Supplementary Information

Supplementary Table S1.
